# Imaging Intracellular Drug/siRNA Co-Delivery by Self-Assembly Cross-Linked Polyethylenimine with Fluorescent Core-Shell Silica Nanoparticles

**DOI:** 10.3390/polym14091813

**Published:** 2022-04-28

**Authors:** Ruirui Zhang, Shuang Wei, Leihou Shao, Lili Tong, Yan Wu

**Affiliations:** 1 Beijing Key Laboratory of Ionic Liquids Clean Process, CAS Key Laboratory of Green Process and Engineering, Institute of Process Engineering, Chinese Academy of Sciences, Beijing 100190, China; zhangzhuruirui@hotmail.com (R.Z.); shweiws@163.com (S.W.); 2 College of Chemistry, Chemical Engineering and Materials Science, Key Laboratory of Molecular and Nano Probes, Ministry of Education, Shandong Provincial Key Laboratory of Clean Production of Fine Chemicals, Shandong Normal University, Jinan 250014, China; lilitong@sdnu.edu.cn; 3 CAS Key Laboratory for Biomedical Effects of Nanomaterials and Nanosafety, CAS Center for Excellence in Nanoscience, National Center for Nanoscience and Technology, Beijing 100190, China; shaoleihou@bcpca.ac.cn

**Keywords:** mesoporous silica, disulfide cross-linked PEI, imaging, fluorescence

## Abstract

Multifunctional theranostic nanomaterial represents one type of emerging agent with the potential to offer both sensitive diagnosis and effective therapy. Herein, we report a novel drug/siRNA co-delivery nanocarrier, which is based on fluorescent mesoporous core-shell silica nanoparticles coated by cross-linked polyethylenimine. The fluorescent mesoporous core-shell silica nanoparticles can provide numerous pores for drug loading and negative charged surface to assemble cross-linked polyethylenimine via electrostatic interaction. Disulfide cross-linked polyethylenimine can be absorbed on the surface of silica nanoparticles which provide the feasibility to bind with negatively charged siRNA and release drug “on-demand”. In addition, the hybrid nanoparticles can be easily internalized into cells to realize drug/siRNA co-delivery and therapeutic effect imaging. This work would stimulate interest in the use of self-assembled cross-linked polyethylenimine with fluorescent mesoporous core-shell silica nanoparticles to construct multifunctional nanocomposites for tumor therapy.

## 1. Introduction

Combination therapy with an anticancer drug and siRNA holds great potential for cancer therapy, which can achieve synergistic effects and overcome the drawbacks of using a single drug, including drug resistance, low efficacy and high toxicity [1,2]. To achieve a maximal effect, the chemotherapeutic drug and siRNA should be delivered into the same tumor cells simultaneously with systemic administration. Recent advances in nanotechnology have offered new opportunities for designing various nanocarriers to co-deliver drug/siRNA [3]. However, most delivery systems lack a suitable signal for long-term and real-time imaging of the process of drug/siRNA delivery as well as the therapeutic effects, which cannot satisfy an evergrowing demand for the improved therapy protocols [4,5,6]. For the assessment of combinational therapy in details, it should be of particular importance to analyze the drug/siRNA delivery process, such as cellular uptake and release behavior of the drug/siRNAs.

Therefore, the integration of imaging agents with drug/siRNA into one nanoplatform for simultaneous intracellular tracking and therapy can provide an advantageous approach in the study of tumor treatment [2,7]. Because the fluorescence technique is one of the most useful analytical tools in bioimaging, a great number of multifunctional therapeutic systems have been fabricated using appropriate fluorescent imaging agents, including organic fluorescent dyes, carbon dots, and quantum dots [8,9]. However, fluorescent dyes usually suffer from the poor photostability and aggregation-caused quenching [10]. These limitations can be overcome by using quantum dots and carbon dots which have been reported as imaging agents due to their unique optical properties [11,12,13]. Unfortunately, the intrinsic toxicity of quantum dots in oxidative biological environments hinders their further biomedical application [12]. And the carbon dots often show a relatively low quantum yield [14]. These facts provide the motivation for designing new materials for fluorescence imaging and drug/siRNA delivery simultaneously. 

Lately, much effort has been devoted to the fabrication of multifunctional nanoparticles based on silica nanoparticles [15,16]. Due to its unique features such as uniform and tunable pore structure, and great diversity in functionalization, silica has been employed as a versatile and useful solid support for constructing various hybrid materials in biomedicine [17,18,19]. In particular, mesoporous silica (MS) with small pores (2–3 nm) has drawn more attention for their distinctive characteristics such as low density, large surface area, and high guest-loading capacity [20,21,22]. To fulfill the needs of cancer therapy, silica nanoparticles should be straightforward prepared and ‘intelligent’ enough to overcome the in vivo biological barriers, and be able to deliver drugs and imaging agents to diseased tissues efficiently [23,24,25]. Among them, one promising strategy is the development of hybrid silica nanoplatforms that integrate imaging and therapeutic modalities [26,27,28]. Moreover, fluorescent labeling of drug carriers can minimizing interference since the fluorophore-modified siRNA can perturb the entry of the siRNAs to the RNAi pathway.

**Scheme 1:**
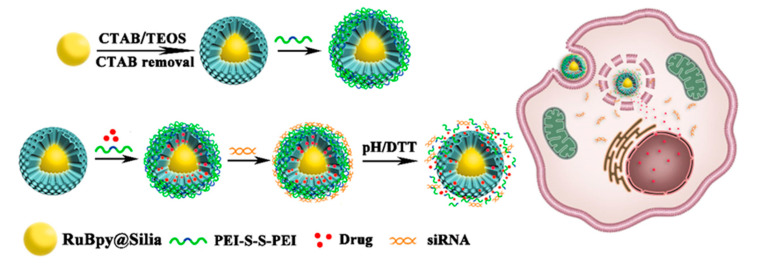
Design of cross-linked polyethylenimine coated fluorescent mesoporous core-shell silica nanoparticles for imaging and drugs/siRNA delivery.

In the present work, we designed and fabricated a drug/siRNA co-delivery nanocarrier that was based on fluorescent mesoporous core-shell silica nanoparticles coated by cross-linked polyethylenimine. The fluorescent mesoporous core-shell silica nanoparticles employed in this study could provide small pores for drug loading and negative charged surface to assemble cross-linked polyethylenimine via electrostatic interaction. Disulfide cross-linked polyethylenimine absorbed on the surface of silica nanoparticles, which provided the feasibility to bind siRNA and release drug “on-demand”. The basic principle of self-assembly cross-linked polyethylenimine with fluorescent mesoporous core-shell silica nanoparticles is shown in Scheme 1.

## 2. Materials and Methods

### 2.1. Materials

Tetraethylorthosilicate (TEOS), tris(2,2′-bipyridine)dichlororuthenium(II) hexahydrate (RuBpy), hexadecyltrimethylammonium bromide (CTAB), di(*N*-succinimidyl) 3,3′-dithiodipropionate (DSP), dithiothreitol (DTT), polyethylenimine (25 kDa branched PEI, Mw ~25,000 Da), polyethylenimine (800 Da branched PEI, Mw ~800 Da), penicillin, and streptomycin were purchased from Sigma-Aldrich (St. Louis, MO, USA). Doxorubicin hydrochloride (Dox) was obtained from Beijing Huafeng United Technology Co., Ltd. (Beijing, China). Cell counting kit-8 was purchased from Dojindo Molecular Technologies (Tokyo, Japan). Dulbecco’s modified Eagle’s medium (DMEM) and fetal bovine serum were purchased from Gibco BRL (Grand Island, NY, USA). Survivin siRNA and Cy3-labeled siRNA were synthesized by Ribo Biochemistry (Guangzhou, China). Anti-survivin antibody and goat anti-rabbit IgG (H + L) HRP were purchased from Bioworld Technology, Co., Ltd. (Nanjing, China). Deionised water (H_2_O) was purified by a Millipore system (Milli-Q, 18.2 MΩ·cm, NANOPure, Barnstead, NH, USA). All other chemicals were from commercial sources and of analytical reagent grade, unless indicated otherwise.

### 2.2. Preparation

#### 2.2.1. Preparation of Disulfide Cross-Linked PEI (dsPEI)

50 mg of PEI (800 Da) was dissolved in 1 mL of DMSO at a final concentration of 50 mg/mL. Next, 5 mg of DSP was dissolved in DMSO (10 mg/mL) and added dropwise to the PEI solution with vortexing. The reaction was allowed to proceed in the dark at room temperature for 2 h, and then the solution was purified by ultrafiltration in an ultrafiltration tube whose cut-off molecular weight was 3 kDa (obtained from Millipore Co., USA) and then lyophilized. The obtained dsPEI stored at 4 °C for further use.

#### 2.2.2. Synthesis of RuBpy Doped Silica (RS) Cores

RuBpy doped silica nanoparticles were synthesized in the *w*/*o* microemulsion system. The microemulsion was consisted of a mixture of 5.3 mL of Triton X-100, 5.4 mL of *n*-hexanol, 22.5 mL of cyclohexane, 3.0 mL of deionized water, 10.0 mg RuBpy, and 0.35 mL of ammonium hydroxide. The microemulsion was stirred for 30 min before 0.3 mL of TEOS was added. The solution was stirred for another 24 h. After the reaction was complete, RS cores were isolated from the microemulsion using acetone, centrifuged and further washed with ethanol, deionized water and *N*,*N*′-dimethyl formamide (DMF) several times to remove surfactant and superfluous dye molecules. The obtained RS nanoparticles were finally redispersed in 9 mL of deionized water for further use.

#### 2.2.3. Synthesis of Mesoporous Core-Shell Silica Nanoparticles (RS@MS)

The prepared RS cores dispersed in water (0.5 mL) were added to a 10.0 mL aqueous CTAB solution (8 mM) under stirring. 0.1 mL of NaOH solution (0.1 M) was subsequently added. Then, 30.0 μL of 20% TEOS in ethanol were injected into the above mixture three times at 30 min intervals. The reaction was allowed to further proceed for 24 h. Finally, the obtained RS@MS nanoparticles were centrifuged and washed with ethanol five times to remove the CTAB molecules.

### 2.3. Characterization and Methods

The structure of each compound was characterized by Fourier-transform infrared spectroscopy (FT-IR) and ^1^H-nuclear magnetic resonance (^1^H-NMR) spectral analysis. For FT-IR analysis, polymer and potassium bromide (KBr) were mixed in a mass ratio of 1:50 to form transparent tablets, and were detected with FT-IR (Perkin-Elmer Inc., Wellesley, MA, USA). For ^1^H-NMR analysis, the products were dissolved in DMSO-d_6_, and analyzed by a Bruker AVANCE 400 NMR spectrometer (Billerica, MA, USA). TEM images were obtained from a JEOL 2100F operated at 200 kV, samples for which were prepared by drying a drop of colloidal solution containing nanoparticles on a copper grid. The average size, polydispersity index (PDI) and zeta potential of nanoparticles were determined by dynamic light scattering (DLS) using a ZetaSizer Nano series Nano-ZS (Malvern Instruments Ltd., Malvern, UK). Determinations were performed at 633 nm with a constant angle of 90° at 25 °C after samples were appropriately diluted in distilled water. Absorption spectra were measured on a TU-1810 UV-vis spectrophotometer (Pgeneral, Beijing, China). Fluorescence spectra were obtained with LS 55 fluorescence spectrometer (Perkin Elmer, Fremont, CA, USA) with a xenon lamp and 1.0 cm quartz cells at the slits of 5.0/5.0 nm. Confocal fluorescence imaging studies were performed with a confocal laser scanning microscopy (Carl Zeiss, Boston, MA, USA).

### 2.4. Tests

#### 2.4.1. Loading, Capping and Release Experiments

RS@MS (10 mg) was stirred in 1 mL of 0.67 mM Dox solution at room temperature for 24 h. Then dsPEI (5 mg) was added to the suspension. The mixture was stirred for another 4 h. The Dox loaded dsPEI-coated RS@MS nanoparticles (RS@MS(Dox):dsPEI) were collected by centrifugation and washed extensively with PBS. The loading content (LC) of Dox was measured using an UV-Vis spectrophotometer at 480 nm. The LC was defined as following formula:

LC = (weight of loaded drug)/(total weight of nanocomposites) × 100%.(1)

For in vitro drugs release study, the RS@MS(Dox):dsPEI were introduced into a dialysis bag (3500 Da), and then dialyzed against 40 mL different PBS solutions (pH 7.4 or pH 5.4) with stirring at 110 rpm/37 °C. At appropriate intervals, the environmental buffer solution was replaced with fresh PBS, and the concentration of the released Dox in the removed PBS was determined by using a UV-Vis spectrophotometer (480 nm).

#### 2.4.2. Cell Culture and Cytotoxicity Assay

Human cervical carcinoma (HeLa) cells were cultured in DMEM medium with 10% FBS, 1% penicillin, and 1% streptomycin, in a 5% CO_2_ incubator at 37 °C. To evaluate the cytotoxicity, HeLa cells were seeded in 96-well plate with a density of 5 × 10^3^ cells per well for 12 h. Then, the original culture medium was replaced by 200 μL fresh culture medium containing various concentrations of PBS, dsPEI, RS@MS:dsPEI and PEI_25000_, Dox, and RS@MS(Dox):dsPEI for 24 or 48 h, respectively. Cell viability was measured by CCK-8 assay, carrying out in accordance with the manufacturer’s instructions (Dojindo, Japan).

#### 2.4.3. siRNA Adsorption

The charge ratio (N/P) of RS@MS:dsPEI-siRNA was indicated as the mole ratio of the tertiary amine groups (N) on dsPEI to the phosphate groups (P) on siRNA. The binding ability of siRNA with RS@MS:dsPEI was evaluated by an agarose gel retardation assay. dsPEI was used as controls. A volume of 10 μL of well-incubated complexes solution containing 1 μg of siRNA were mixed with loading buffer, then the suspensions were loaded onto 1% agarose gel and 5 μg/mL ethidium bromide. The gels were run in 1× TAE buffer at a voltage of 120 V for 15 min and then visualized by a UV illuminator.

#### 2.4.4. Western Blot Analysis

HeLa cells were seeded in a 6-well plate with 1 × 10^5^ cells per well in 2 mL of DMEM medium and cultured for overnight. Then the cells were implemented different operations. After further incubation for 48 h at 37 °C, these cells were washed with PBS for three times and collected, lysed with RIPA buffer. The concentration of denatured proteins was calculated with a BCA Protein Assay Kit. Subsequently, the equal amount of proteins were separated using gel electrophoresis and transferred onto a poly(vinylidene difluoride) (PVDF) membranes. Then, after blocking with 5% (*w*/*v*) milk, the membranes were incubated with primary antibody and secondary antibody, respectively. Finally, protein bands were imaged by a ChemiDoc XR + UV illuminator.

#### 2.4.5. In Vitro Cellular Uptake

HeLa cells were cultured in DMEM media containing 10% FBS and incubated at 37 °C incubator with 5% CO_2_ for 24 h. The original culture medium was then replaced by fresh culture medium containing RS@MS(Dox):dsPEI-siRNA at the same concentration of Dox (5 μM) and the cells were cultured for another 2 or 6 h, respectively. Then the cells were washed with pH 7.4 PBS three times before investigation with confocal laser scan microscopy.

#### 2.4.6. Hemolysis Assay

Hemolysis rate test was determined according to previously reported. The normal RBCs were drawn from the heparin-stabilized blood of the male Wistar rats. 0.2 mL RS@MS(Dox):dsPEI solutions at different concentration were incubated for 60 min in 0.8 mL Normal saline (NS). Then, 0.2 mL RBCs solution was added into 0.8 mL RS@MS(Dox):dsPEI solution and incubated at 37 °C for another 2 h. Positive and negative controls are RBCs in water and saline, respectively. After centrifugation at 3000 rpm for 5 min, the optical density (OD) of the supernatant was read at 545 nm using a UV-vis spectrophotometer. The positive reference (100% lysis) was a blood/water mixture, and the negative reference (0% lysis) was a blood/saline mixture. The hemolytic ratios of the samples were calculated as follows:

(2) $$\mathrm{hemolytic}\;\mathrm{ratio}\;(\%)=\frac{\mathrm{sample}\;\mathrm{absorbance}\;-\;\mathrm{negative}\;\mathrm{control}}{\mathrm{positive}\;\mathrm{control}\;-\;\mathrm{negative}\;\mathrm{control}}$$

## 3. Results and Discussion

### 3.1. Construction and Characterization of RS@MS:dsPEI Nanoparticles

**Figure 1:**
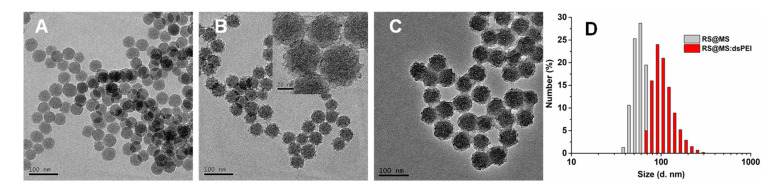
TEM images of (**A**) RS cores, (**B**) RS@MS core-shell nanoparticles, (**C**) RS@MS:dsPEI nanoparticles, and (**D**) particle sizes distribution of RS@MS and RS@MS:dsPEI. The inset in B is a magnified TEM image of the corresponding sample.

Fluorescent mesoporous core-shell silica nanoparticles were synthesized according to a modified report [29]. RS nonporous silica cores were prepared through a typical reverse microemulsion method [30]. Tris(2,2′-bipyridine)dichlororuthenium with good stability and high quantum yield was chosen as the fluorescent dye to doped in the silica core [31]. MS shells were formed by injecting a silica alkoxide precursor to CTAB template surrounded silica cores and then the template molecules were removed by washing with organic solvent. Figure 1A,B showed that the RS cores and RS@MS nanoparticles were uniform and well-dispersed without aggregation. In contrast to the nonporous silica cores, disordered wormhole-like mesopores shell with diameters of around 2–4 nm was observed on the surface of core-shell nanoparticles (the Inset of Figure 1B), which provided sufficient space for drug loading. TEM images showed that both RS@MS and RS@MS:dsPEI nanoparticles with a diameter between approximately 60 and 80 nm, which were smaller than that obtained by DLS (Figure 1D). This difference is attributed to the diameter obtained by DLS reflected the hydrodynamic diameter of nanoparticles swelled in aqueous solution, while those observed by TEM was the diameters of dried nanoparticles.

**Figure 2:**
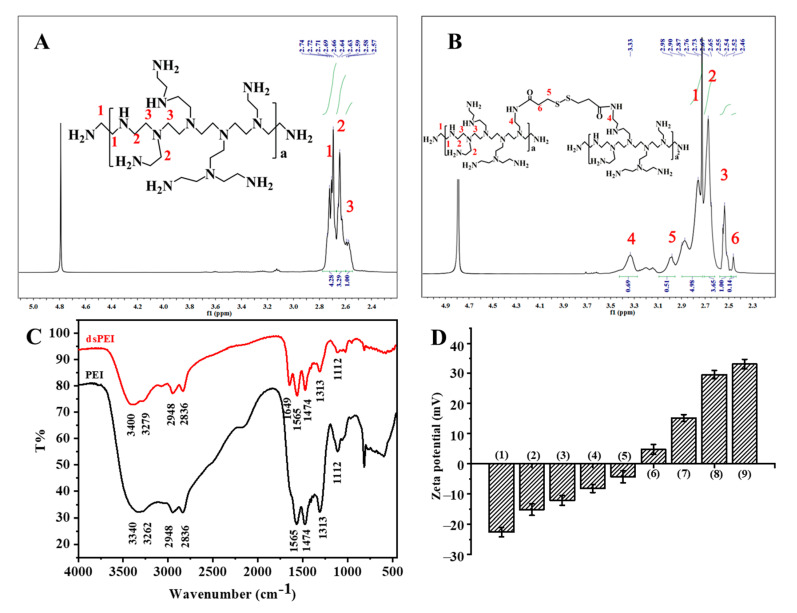
(**A**) ^1^H NMR spacetra of PEI. (**B**) ^1^H NMR spacetra of dsPEI. (**C**) FT-IR spectra of PEI and dsPEI. (**D**) Zeta-potential measurements (1) RS, (2) RS@MS, (3) RS@MS:dsPEI (*w*/*w* 1:0.25), (4) RSMS:dsPEI (*w*/*w* 1:0.5), (5) RS@MS:dsPEI (*w*/*w* 1:1), (6) RS@MS:dsPEI (*w*/*w* 1:1.5), (7) RS@MS:dsPEI (*w*/*w* 1:2), (8) RS@MS:dsPEI (*w*/*w* 1:4), (9) RS@MS:dsPEI (*w*/*w* 1:10), all the samples were dissolved or suspended in deionized water.

Disulfide cross-linked PEI (dsPEI) was achieved through the reaction of crosslinking reagent DSP with the low molecular PEI. The chemical structure of dsPEI was confirmed by ^1^H NMR and FT-IR. Comparing of Figure 2A, the peak at 2.4 and 3.0 ppm were assigned to the protons of CH_2_ in DSP (Figure 2B). As shown in Figure 2C, compared with the FT-IR spectrum of PEI, the characteristic peaks of CH_2_ (2948 cm^−1^ for asymmetric stretching vibration; 2836 cm^−1^ for symmetric stretching vibration; 1474 cm^−1^ for scissor bending vibration), NH_2_ (1565 cm^−1^ for scissor bending vibration) and C-N (1313 cm^−1^ and 1112 cm^−1^ for stretching vibration) could also be found in that of dsPEI. The NH_2_ symmetric/asymmetric stretching vibration peaks from dsPEI at 3279 cm^−1^ and 3400 cm^−1^ showed slightly shift relative to that of PEI at 3262 cm^−1^ and 3340 cm^−1^, which could be ascribed to the formation of amide bond between NH_2_ and DSP. Additionally, an emerging stretching vibration of C=O bonds belonged to the crosslinker DSP at 1649 cm^−1^ in the spectrum of dsPEI, further confirmed the successful construction of dsPEI polymer [32]. Due to the negative zeta potential of the silica nanoparticles, polycationic PEI molecules can be attached to nanoparticle surfaces through electrostatic interactions [33,34]. The reversed zeta-potential value of the RS@MS:dsPEI complexes indicated the successful coating of dsPEI onto the RS@MS surfaces (Figure 2D) and thick polymer coating was observed around the silica particle, which could be further confirmed by the TEM image from Figure 1C. Upon increasing the dsPEI amount, the RS@MS:dsPEI complexes showed increased positive zeta potential values, and the zeta-potential value of RS@MS:dsPEI was determined to be 16 mV at the mass ratio of 1/2 (RS@MS/dsPEI), meaning that the RS@MS:dsPEI could be as gene carriers.

**Figure 3:**
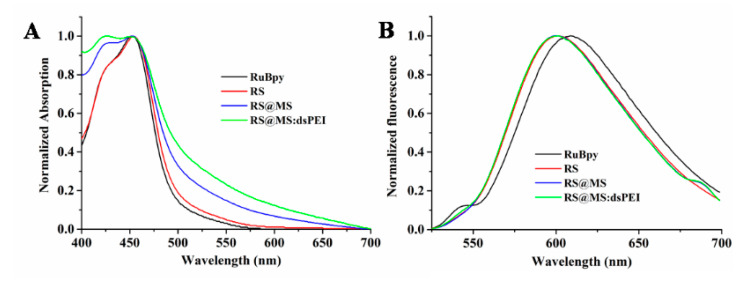
(**A**) Normalized absorption spectra of RuBpy, RS, RS@MS, and RS@MS:dsPEI. (**B**) Normalized fluorescence emission spectra of RuBpy, RS, RS@MS, and RS@MS:dsPEI.

As can be seen from Figure 3A, RuBpy, RS, RS@MS, and RS@MS:dsPEI all displayed typical absorption spectra of RuBpy. The fluorescent spectra taken from nanoparticles were slightly shifted to the blue region compared with the emission spectrum of free RuBpy solution, which due to the dyes were slightly aggregated and had been encapsulated in the silica cores (Figure 3B) [31]. This assembly was not only provided imaging agents but also utilized silica nanoparticles as a connector which could further absorb positive dsPEI by electrostatic interaction. In addition, the loading capacity of the anticancer drug Dox was also determined, and the Dox loading capacity of the RS@MS:dsPEI was measured to be 3.6 wt% of RS@MS. And the encapsulation efficiency of Dox is dependent on loading concentration, when the content of Dox is 0.1 mg/mL, the encapsulation efficiency is about 37.5%. 

### 3.2. In Vitro Dox Release of RS@MS:dsPEI Nanoparticles

**Figure 4:**
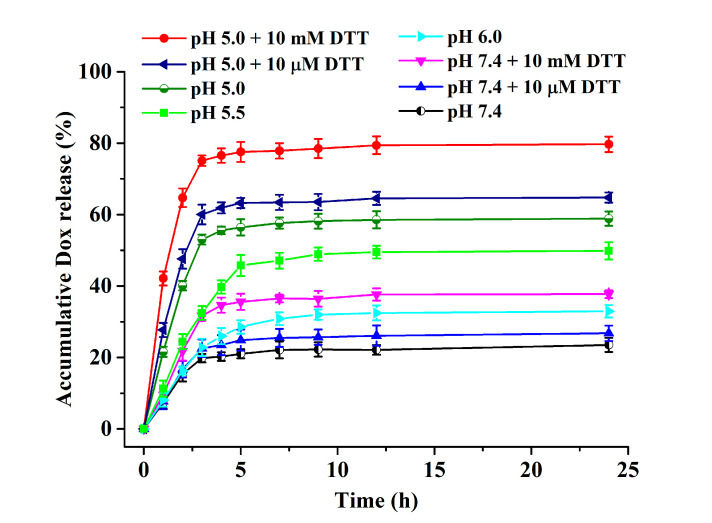
In vitro drug release profiles of Dox from RS@MS(Dox):dsPEI nanoparticles in PBS at different pH with or without DTT, respectively, at 37 °C.

The difference of pH and glutathione (GSH) concentration offer several chances for constructing the “on-demand” delivery system of cancer therapy. The extracellular environment of tumor tissues is more acidic than systemic blood pH and GSH is present in the intracellular matrix of cancer cells at levels two to three orders of magnitude higher than that found in extracellular environments [35]. To explore whether RS@MS:dsPEI exhibited pH/redox dual-responsive intracellular drug release profile, Dox release from the nanoparticles was investigated in PBS containing various concentrations of dithiothreitol (DTT) at various pH values. As shown in Figure 4, for a pH of 7.4 without DTT, less than 22% Dox was released in a time period of 24 h, but in the presence of 10 μM DTT, the values slightly increased to 25%. However, Dox release from the nanoparticles at pH 5.0 was dramatically enhanced compared to that at pH 7.4 in the presence or absence of the same DTT concentration. Dox release from the nanoparticles reached as high as 80% in PBS containing 10 mM DTT at pH 5.0, evidencing effective Dox release under mimic intracellular reducing environment. In addition, as the pH of the external environment changes from 7.4, 6.0, 5.5 to 5.0, the rate of drug release accelerates. These results clearly indicated that the RS@MS:dsPEI nanoparticles could realize “on-demand” drug release by simultaneous pH-induction or reduction activation, which showed promising applications in site-specific tumor therapy.

### 3.3. Cell Cytotoxicity

**Figure 5:**
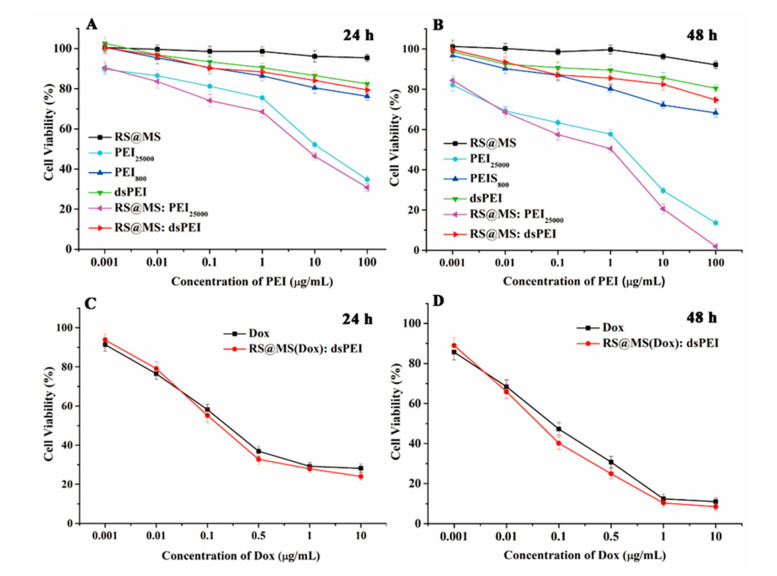
(**A**,**B**) in vitro cytotoxicity of RS@MS, PEI_25000_, PEI_800_, dsPEI, RS@MS:PEI_25000_, and RS@MS:dsPEI nanoparticles against HeLa cells with 24 h and 48 h, respectively; (**C**,**D**) in vitro antitumor activity of Dox and RS@MS(Dox):dsPEI nanoparticles against HeLa cells with 24 h and 48 h, respectively.

The cytotoxicity of RS@MS, PEI_25000_, PEI_800_, dsPEI, RS@MS:PEI_25000_ and RS@MS:dsPEI were tested in HeLa cells using CCK-8 assay (Figure 5). The viabilities of HeLa cells treated with RS@MS nanoparticles were all above 90%, showing that these nanoparticles were not inherent cytotoxic for the cells. Low molecular weight of PEI (800 Da, PEI_800_) revealed significantly lower cytotoxicity compared to high molecular weight of PEI (25 kDa, PEI_25000_), and the cross-linked PEI_800_ showed minimized cytotoxicity. However, RS@MS complexed with dsPEI and PEI_25000_ exhibited higher cytotoxicity due to the positive charge of surface, which in turn promoted internalization into the cell [36]. The cytotoxicity of Dox loaded RS@MS:dsPEI (RS@MS(Dox):dsPEI) also showed an obvious concentration dependent effect (Figure 5C,D). Meanwhile, RS@MS(Dox):dsPEI showed similar cytotoxic effects to HeLa cells compared with free Dox, which might be due to the successful Dox release from RS@MS:dsPEI nanoparticles in response to lysosomal pH- and redox-responsive intracellular microenvironment.

### 3.4. siRNA Binding Capacity and Drug/siRNA Co-Delivery In Vitro

**Figure 6:**
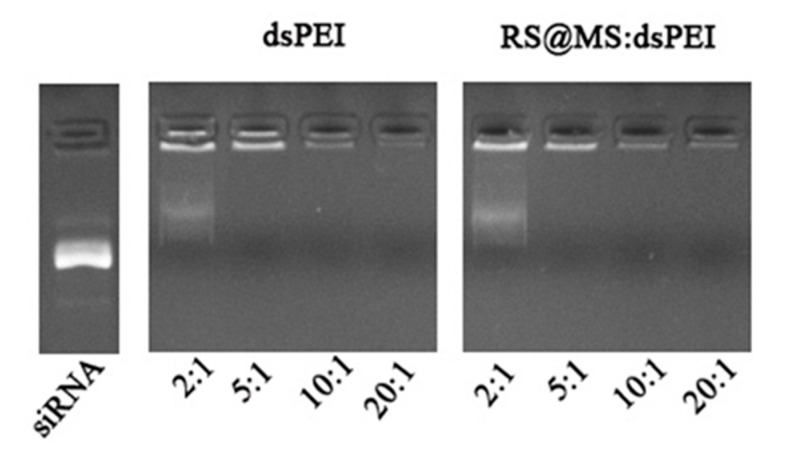
Agarose gel electrophoresis assay of PEI_25000_/siRNA dsPEI/siRNA, and RS@MS:dsPEI/siRNA at different N/P ratios.

To prove the superiority of the present RS@MS:dsPEI nanoparticles, siRNA binding capability measurements were carried out. As shown in Figure 6, the agarose gel electrophoresis assay indicated that RS@MS:dsPEI has efficient siRNA binding capability and could completely retard siRNA migration at N/P ratio of 5. Moreover, the loading of the anticancer drug Dox did not affect the siRNA binding capability of the RS@MS:dsPEI, which allowed the drug/siRNA co-delivery for the following studies [37].

**Figure 7:**
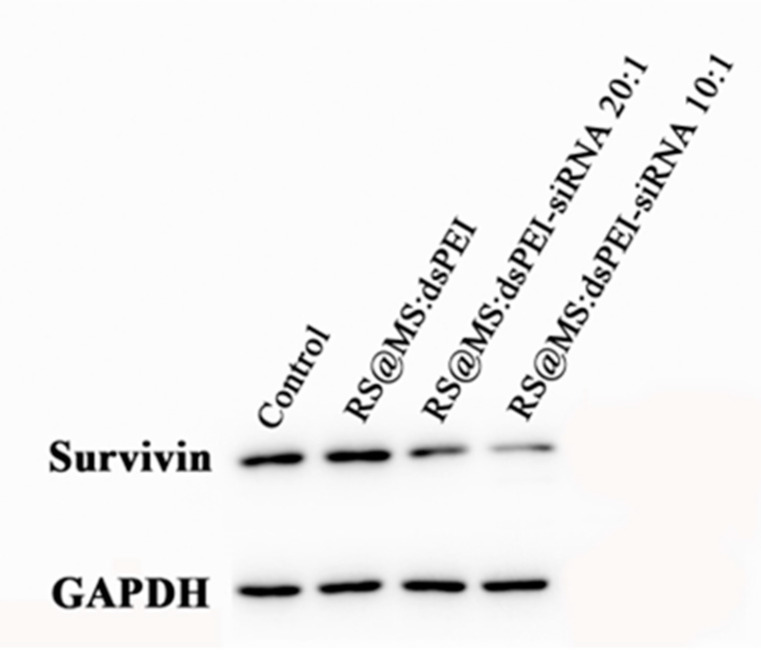
Western-blot assay. HeLa Cells were treated by RS@MS:dsPEI, RS@MS:dsPEI-siRNA with N/P ratios of 10:1 and 20:1, respectively.

The silencing efficiencies of survivin gene by different formulations were evaluated by Western-blot analysis. The expression of survivin protein was dramatically down-regulated by RS@MS:dsPEI/siRNA at different N/P ratios, confirming the intracellular delivery and gene silencing efficiency of surviving siRNA by this nanocarrier (Figure 7).

**Figure 8:**
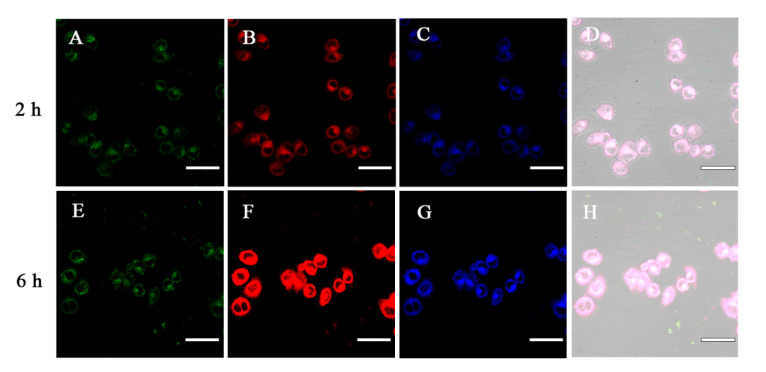
Confocal microscopy images of HeLa cells incubated with RS@MS(Dox):dsPEI-siRNA nanoparticles for 2 h (**A**–**D**) and 6 h (**E**–**H**) at 37 °C, respectively. The RS@MS was recorded in the green channel with excitation at 405 nm; Dox was recorded in the red channel with excitation at 488 nm; Cy3-siRNA was recorded in the blue channel with excitation at 543 nm. Scale bar = 50 μm.

Confocal laser scanning microscopy (CLSM) was applied to detect cellular uptake and distribution of the RS@MS(Dox):dsPEI-siRNA nanoparitlces in HeLa cells. Significant stronger fluorescent signals were observed in HeLa cells treated with RS@MS(Dox):dsPEI-siRNA, indicating the enhanced internalization and/or stability. As can be seen from Figure 8, after HeLa cells were incubated with RS@MS(Dox):dsPEI-siRNA, a strong green fluorescence signal for RuBpy, a blue fluorescence signal for Cy3-labeled siRNA, and a red fluorescence signal for Dox were observed. These three fluorescence signals overlapped mostly at first, indicating that the nanoparticles mostly accumulated in the cytoplasm and the fluorescent silica cores could act as a fluorescent probe for tracing the cellular uptake and intracellular kinetics of the drug/siRNA delivery in real time. Moreover, CLSM imaging also revealed that the red fluorescence derived from Dox was clearly visible in the cytoplasm after 2 h treatment. While, after 6 h treatment, Dox exhibited efficient intracellular delivery in tumor cells and partly entered into the nucleus, which suggested a pH- and redox- dependent drug release profile within cells. It should be noted that once the drug was released from the pore, the fluorescence would be enhanced significantly. That attributed to the aggregation of Dox in the silica pores, which led to the fluorescence self-quenching. In addition, as time went on, the blue fluorescence strengthened which suggested that the uptake increased and partially separated from dsPEI, pointing to the successful escape of Cy3-siRNA and distribution in the cytoplasm where siRNA mediated its function [38]. However, there was only a slight difference in the fluorescence intensity of the nanocarriers after 2 h of treatment and 6 h of treatment. These results further confirmed that the present nanocarrier could effectively deliver the drugs/siRNA into living cells.

### 3.5. Hemocompatibility of RS@MS(Dox):dsPEI Nanoparticles

**Figure 9:**
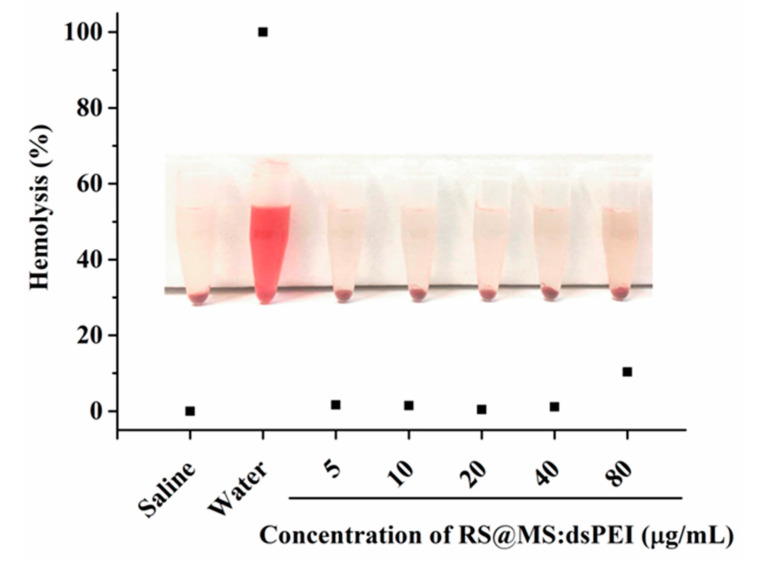
Hemolysis assay of RS@MS(Dox):dsPEI, using water as positive control and saline as a negative control.

To better define the biocompatibility of RS@MS(Dox):dsPEI nanoparticles, a hemolysis assay was conducted to evaluate its compatibility in blood. According to the ISO/TR 7405-1984 (f), nanoparticles were considered of hemolytic if the extent of hemolysis via the quantitation of free hemoglobin in the mixed solution is higher than 5%. As shown in Figure 9, almost no hemolysis of the RBCs could be detected at the RS@MS(Dox):dsPEI concentrations from 5–40 μg/mL, and only slight hemolysis could be observed when the concentration reached to 80 μg/mL. These results indicated that RS@MS(Dox):dsPEI nanocarriers had a good blood compatibility.

## 4. Conclusions

In conclusion, we have described a drug/siRNA co-delivery nanocarrier that is based on cross-linked polyethylenimine coated fluorescent mesoporous core-shell silica nanoparticles. It has shown that the fluorescent silica cores can act as a fluorescent probe for tracing the cellular uptake and intracellular kinetics of the drug/siRNA delivery in real time. The nanopores of silica can be loaded different chemo-drugs and assembled with cross-linked polyethylenimine via electrostatic interaction to provide the feasibility to bind negatively charged siRNA. In addition, disulfide cross-linked polyethylenimine can be absorbed on the surface of silica nanoparticles which realize pH- and redox-responsive synergistic controlled release. Moreover, the nanoparticles can be easily internalized into cells to realize drug/siRNA co-delivery and therapeutic effect. These results make the hybrid nanoparticles reported here as a promising candidate in cancer treatment research.

## Data Availability

Not applicable.
